# Complication of Early Application of One and a Half Hip Spica for Femoral Shaft Fractures in Children below Six Years in a Tertiary Care Hospital: A Descriptive Cross-sectional Study

**DOI:** 10.31729/jnma.6779

**Published:** 2021-10-31

**Authors:** Poojan Kumar Rokaya, Mangal Rawal, Tufan Singh Kathayat, Abhishek Kumar Thakur, Monika Lama, Ramu Maharjan

**Affiliations:** 1 Department of Orthopedics and Trauma Surgery, Karnali Academy of Health Sciences, Jumla, Karnali, Nepal; 2 School of Nursing, Karnali Academy of Health Sciences, Jumla, Karnali, Nepal

**Keywords:** *children*, *early*, *femur*, *fracture*, *hip spica*

## Abstract

**Introduction:**

Hip spica with or without prior traction has become a standard treatment for closed isolated femoral shaft fracture in children below six years. The time of hip spica application is not uniform in the existing literature. The aim of this study is to find out the prevalence of complication of early application of one and a half hip spica for femoral shaft fractures in children below six years in a tertiary care hospital.

**Methods:**

A descriptive cross-sectional study was done among 42 children who were managed with early hip spica application (within 72 hours) for femoral shaft fracture from January 2015 to December 2020 after receiving ethical clearance from Institutional Review Committee. Convenient sampling technique was done. Point estimate at 95% Confidence Interval was calculated along with frequency and proportion for binary data.

**Results:**

Complication was seen in nine (21.4%) (95% Confidence Interval= 9.02-33.84) patients. Skin breakdown from local pressure had the highest prevalence in our study which was documented in three (7.14%) patients who resolved with conservative treatment.

**Conclusions:**

The prevalence of complication of early application of one and a half hip spica for femoral shaft fractures in children below six years was comparable to the similar studies done in similar setting.

## INTRODUCTION

Femoral shaft fracture is common in children. They represent 1.6% of all childhood fractures.^1^ Widely accepted treatment methods include hip spica application, external fixation, flexible intramedullary nailing, rigid intramedullary rod, and plate fixation.^2^

Hip spica with or without prior traction has become a viable treating option for closed isolated femoral shaft fracture in children below six years.^3^ The time of hip spica application varies from immediate (within 24 hours) to early (within 72 hours)to late (10-12 days after skin traction).^4-10^ Early hip spica application is associated with potential complications like excessive shortening, peroneal nerve palsy, angular deformity, and loss of reduction.^11-12^ Late hip spica application predisposes to skin ulceration, pin tract infection, prolonged hospital stay, and costly treatment.^5,6^ The time of hip spica application and its outcome is not uniform in the existing literature.^6,9,11^

The aim of this study is to find outthe prevalence of complications of early application of one and a half hip spica for femoral shaft fractures in children below six years in a tertiary care hospital.

## METHODS

A descriptive cross-sectional study was conducted after ethical clearance (IRC KAHS Reference: 17/077/078). All the children who presented to Karnali Academy of Health Sciences, Jumla with femoral shaft fractures from January 2015 to December 2020 were identified by the medical records. Children from birth to six years of age managed with one and a half hip spica for femoral shaft fracture within 72 hours of injury were included in the study. Exclusion criteria were children above six years of age, pathological fracture, femur physeal injury, intra-articular fracture, neck of femur fracture, and patients with incomplete clinical and radiological details.

Sample size calculation was done using the formula,

n = Z^2^ × p × q / e^2^

  = (1.645)^2^ × 0.5 × 0.5 / (0.13)^2^

  = 40

where,

n = sample size
Z = 1.645 at 95% confidence interval
p = prevalence taken as 50%
q = 1 - p
e = margin of error, 13%.

The sample size calculated was 40. However, 42 samples were taken in the study. Initially, the fractured limb was supported with an appropriate splint. One and a half hip spica was applied in the plaster room under conscious sedation after manual reduction by registered orthopedic surgeons. Hip spica incorporated the torso starting from the level of nipples, abdomen, pelvis, hip, thigh, knee, leg, and ankle of the affected side and hip and thigh of uninjured side with a wooden crossbar in between. The optimal position of joints inside the hip spica included bilateral hip abduction of 30-45°, hip flexion 30-40°, kneeflexion 60-75°, and leg in 15° of external rotation. Hip flexion on the affected side was increased for fracture involving the proximal third of the femoral shaft. Hip spica was well molded with special attention to valgus mold at the fracture site to prevent varus deformity. Hip spica ends were carefully trimmed and adequate padding was applied to avoid point pressure and plaster sore. Enough space was left for perineal toileting. A standard radiograph of the injured thigh was ordered to assess the fracture reduction. Children were discharged after the hip spica was completely dry. Potential plaster complications and hip spica care were explained to the parents.

For the first two weeks, patients were followed up every week to evaluate the fracture and plaster complication. Then after patients were followed up every fortnight until the radiological union was achieved. Fracture union was defined as the radiological appearance of the bridging femoral cortices on at least three of four cortices on the anteroposterior and lateral view.^13^

During a routine follow up cast wedging or spica change was done as per need. Once the radiological union was evident spica was removed on a daycare basis and physiotherapy commenced. Protected weight bearing and gentle range of motion exercise of bilateral hip and knee were advised. Patients were followed for at least six months from injury to assess residual clinical and radiological deformity.

Clinically, limb length discrepancy, limb alignment, and knee range of motion were assessed. Limb length was measured in the supine position from the anterior superior iliac spine of the squared pelvis to the tip of the medial malleolus with measuring tape and skin marker. Length inequality of more than two cm compared to the normal contralateral limb was considered significant.^9^ The range of motion of the affected knee joint was measured manually using a goniometer. Standard anteroposterior and lateral view of the entire femur with 100% magnification was obtained to assess fracture shortening, varus and valgus angulation in the frontal plane, posterior angulation (recurvatum) ,and anterior angulation (procurvatum) in the sagittal plane. Angular deformity of > 15° in the frontal plane and >20° in the sagittal plane was defined as malunion. Incomplete osseous fusion within 3 months from injury was defined as delayed union whereas absence of osseous fusion more than six months after the injury was defined as nonunion.^14^

All the pertinent sociodemographic information, clinical and radiological data were collected and entered in individual patient proforma. Data were entered in MS EXCEL and transferred to statistical software statistical package for social sciences version 16. Descriptive statistics like number, percentage, mean, average and range was used to describe the variables.

## RESULTS

In this study, 42 children met the inclusion criteria. The complication was seen in nine (21.43%) (95% Confidence Interval= 9.02-33.84) patients. All the complications were minor (Table 1).

**Table 1:** Types of complication.

Complication	n (%)
Skin breakdown	3 (7.14)
Cast wedging	2(4.76)
Hip spica re-application	2(4.76)
Varus angulation	1(2.38)
Shortening	1(2.38)

Skin breakdown from local pressure was documented in three patients who resolved with conservative treatment. Cast wedging was required in two patients with unacceptable varus angulation. Two patients with hip spica breakage and soakage from poor hygiene underwent removal and reapplication of a spica. In the last follow up there was an average shortening of 8.4 mm whereas one patient was observed to have 24 mm of limb shortening. Angular deformity and limb length inequality were considered minor because none of these problems required further surgical intervention. The mean range of motion of the affected knee joint was 134.4° (Range 116-154°). The mean age was 3.6 years (8months- 6 years). Patient and fracture characteristics are shown in table 2 (Table 2).

**Table 2:** Patient and fracture characteristics.

Variables		n (%)
Gender	Boys	28 (66.6)
	Girls	14(33.3)
Side	Right	25 (60)
	Left	17(40)
Mode of injury	Fall from ladder	15 (35.7)
	Fall from rooftop	9 (21.4)
	Sports	7 (16.6)
	Fall from bed	5 (11.9)
	Road traffic accidents	4 (9.5)
	Fall from bicycle	2 (4.7)
Fracture Type	Spiral	20 (47.6)
	Transverse	12 (28.5)
	Long Oblique	6 (14.2)
	Short Oblique	4 (9.5)
Fracture Location	Proximal third	10 (23.8)
	Middle third	28 (66.6)
	Distal third	4 (9.5)
	Mild head injury	2 (4.7)
Associated Injuries	Ipsilateral distal physeal injury Pubic Rami fracture Contralateral Iliac fracture	radius Blade	1 (2.3) 1 (2.3) 1 (2.3)

Residual angular deformity (varus 18°) was noted in one patient. None of the patients with residual angular deformity and limb length discrepancy complained of pain or functional limitation. Radiological fracture shortening and angulation at injury and final follow-up are summarized in Table 3 and Table 4 respectively (Table 3, Table 4).

**Table 3:** Radiographic parameters at injury (n = 42).

Variable	Shortening	Varus	Valgus	Recurvatum	Procurvatm
	(mean ±SD)	(mean ±SD)	(mean ±SD)	(mean ±SD)	(mean ±SD)
Average	18±2.8mm	15±4.6°	9±2.0°	12±3.78°	10±2.56°
Range	0-38mm	5-50°	3-18°	4-38°	2-20°

**Table 4:** Radiographic parameters at final follow up (n = 42).

Variable	Shortening	Varus	Valgus	Recurvatum	Procurvatm
	(mean±SD)	(mean±SD)	(mean±SD)	(mean±SD)	(mean±SD)
Average	8.4±2.18mm	7±3.88°	4±1.17°	5±0.98°	3±1.03°
Range	0-24mm	3-18°	2-10°	1-12°	1-10°

There were no nonunion. Neuropraxia or compartment syndrome after hip spica application was not found. The average duration of presentation to the hospital was 2.2 days (Range 1-6 days). The mean length of hospital stay was 2.8 days (Range1-4 days). The average duration of hip spica immobilization was six weeks (Range 5 to 8 weeks). The mean follow-up duration was nine months (Range 6-36 months).

## DISCUSSION

Femur fractures are one of the most common childhood injuries requiring inpatient admission. The main goal of treatment is to achieve timely union with acceptable length, rotation and alignment. Most of our patients belong to low socio-economic backgrounds, conservative management is commonly practiced in our setup. Our teaching hospital treatment protocol for childhood femur fracture is individualized on age, body habitus, fracture stability and location, skin condition and associated injures. We have been treating femoral shaft fractures in children below six years of age with early hip spica which is supported by various studies that advocate immediate to early hip spica for the treatment of femoral shaft fractures in children.^15-17^

This study reflects the outcome and complications of early hip spica application. Most of the complications were minor and plaster-related. We noted skin breakdown from local pressure of hip spica plaster in three patients which improved with skincare. Jauquier et al and Defazio et al reported similar complications limited to hip spica plaster.^6,18^ Extra padding and proper trimming of the cast ends can minimize these minor plaster related complications. One of the advantages of hip spica application is the provision of cast wedging when fracture becomes displaced or misaligned. Two of our patients in the first week follow-up underwent valgus wedging to correct unacceptable varus angulation. A retrospective study by Younis et al reported wedging of spica in three patients who were treated with early hip spica for femoral shaft fractures."Varus angulation in the frontal plane is a common deformity after femoral shaft fracture which is preventable by applying a well molded hip spica with special valgus mold at the fracture site.

Hip spica was reapplied in two patients who had spica breakage and soiling. Mansour et al mentioned hip spica breakage and soiling in six patients who required hip spica change.^5^Younis et al reported hip spica change in three patients who were treated with early hip spica for femoral shaft fractures.^11^ Parents should be properly counseled regarding hip spica care, handling of hip spica, perineal toileting and skincare. We have noted that improper handling of hip spica through the wooden crossbar leads to breakage of hip spica. In our study, all the fractures consolidated in an acceptable position in both the frontal and sagittal plane except for one patient who had residual varus angulation of 18°. Our acceptable radiological results are supported by prior studies of immediate to early hip spica application for femur fractures in children.^8,9,11^ Due to predictable remodeling capacity in younger children up to 20° of femoral shaft malangulation can be tolerated. The average shortening in this series was 8.4 mm. One patient in the last follow-up was measured to have a shortening of 24 mm but his parents did not complain of limp or functional limitation. Berne, et al. described limb length discrepancy of more than 10mm in five children while Jamaludin, et al. described an average shortening of 9.5mm which is comparable to our study.^19^,^20^ We observed good results and favorable clinical and radiological outcomes after early hip spica application for femoral shaft fractures in children below six years.

Descriptive cross-sectionalstudy with a small sample size was one of the few limitations of this study. We did not seek risk factors responsible for developing complications after hip spica application. Our follow-up duration was short which may not justify the final residual deformity as bone remodeling can be expected to occur till the skeletal maturity.

## CONCLUSIONS

The prevalence of complication of early application of one and a half hip spica for femoral shaft fractures in children below six years was comparable to the similar studies done in similar setting.
